# Online Social Networks That Connect Users to Physical Activity Partners: A Review and Descriptive Analysis

**DOI:** 10.2196/jmir.2674

**Published:** 2014-06-16

**Authors:** Atul Nakhasi, Album Xiaotian Shen, Ralph Joseph Passarella, Lawrence J Appel, Cheryl AM Anderson

**Affiliations:** ^1^School of MedicineJohns Hopkins UniversityBaltimore, MDUnited States; ^2^Johns Hopkins UniversityBaltimore, MDUnited States; ^3^School of MedicineDepartment of Family and Preventive MedicineUniversity of California, San DiegoLa Jolla, CAUnited States

**Keywords:** behavior, behavior control, behavioral research, exercise, health, health behavior, information services, Internet, intervention studies, online systems, physical fitness, social media, social medicine, social networking, social support, telemedicine, Web

## Abstract

**Background:**

The US Centers for Disease Control and Prevention have identified a lack of encouragement, support, or companionship from family and friends as a major barrier to physical activity. To overcome this barrier, online social networks are now actively leveraging principles of companion social support in novel ways.

**Objective:**

The aim was to evaluate the functionality, features, and usability of existing online social networks which seek to increase physical activity and fitness among users by connecting them to physical activity partners, not just online, but also face-to-face.

**Methods:**

In September 2012, we used 3 major databases to identify the website addresses for relevant online social networks. We conducted a Google search using 8 unique keyword combinations: the common keyword “find” coupled with 1 of 4 prefix terms “health,” “fitness,” “workout,” or “physical” coupled with 1 of 2 stem terms “activity partners” or “activity buddies.” We also searched 2 prominent technology start-up news sites, TechCrunch and Y Combinator, using 2 unique keyword combinations: the common keyword “find” coupled with 1 of 2 stem terms “activity partners” and “activity buddies.” Sites were defined as online social health activity networks if they had the ability to (1) actively find physical activity partners or activities for the user, (2) offer dynamic, real-time tracking or sharing of social activities, and (3) provide virtual profiles to users. We excluded from our analysis sites that were not Web-based, publicly available, in English, or free.

**Results:**

Of the 360 initial search results, we identified 13 websites that met our complete criteria of an online social health activity network. Features such as physical activity creation (13/13, 100%) and private messaging (12/13, 92%) appeared almost universally among these websites. However, integration with Web 2.0 technologies such as Facebook and Twitter (9/13, 69%) and the option of direct event joining (8/13, 62%) were not as universally present. Largely absent were more sophisticated features that would enable greater usability, such as interactive engagement prompts (3/13, 23%) and system-created best fit activities (3/13, 23%).

**Conclusions:**

Several major online social networks that connect users to physical activity partners currently exist and use standardized features to achieve their goals. Future research is needed to better understand how users utilize these features and how helpful they truly are.

## Introduction

 In 2006, it was estimated that direct medical costs in the United States because of a lack of physical activity totaled more than $188 billion annually [1]. The US Centers for Disease Control and Prevention (CDC) have identified a lack of encouragement, support, or companionship from family and friends as a major barrier to physical activity [2]. Online social networks are now actively leveraging principles of companion social support in novel ways to overcome this barrier. For example, services that facilitate step-count sharing between friends and allow users to engage in fitness challenges with one another by sharing workout routines have recently emerged [3]. Users have embraced physical activity social networks, such as Fitocracy, Spark People, and Run Keeper, which currently have an estimated 1 million, 15 million, and 23 million users, respectively [4-6].

With the boom of online social networks focused on physical activity, new sites that directly connect individuals to physical activity partners have emerged. The social support provided through the discussion forums of these networks and the ability to connect with users online likely increases physical activity; the existing literature supports that having one friend to exercise with increases the likelihood of doing so by 45% [7]. As the industry for online physical activity social networks grows, it would be helpful to characterize existing networks and examine approaches taken by sites to leverage social support to increase physical activity engagement.

As a first step toward understanding the potential that online social networks have to increase physical activity and in an effort to inform the development of future networks, we aim to (1) describe established and emerging social networks that connect users to physical activity partners face-to-face, and (2) describe the functionality, features, and usability of these networks.

## Methods

### Identification of Established and Emerging Social Health Activity Networks

To identify the established and emerging social health activity networks, we searched 3 major databases: the Google search engine and 2 start-up news sites, TechCrunch [8] and Y Combinator [9]. TechCrunch and Y Combinator comprehensively cover online social networks, and these 3 sites were selected with the expectation that they covered social networks with sufficient popularity and exposure. Initially, we identified established networks using a Google search for the top 30 results from use of the common keyword “find” coupled with 1 of 4 different prefix terms “health,” “fitness,” “workout,” or “physical” coupled with 1 of 2 different stem terms “activity partners” or “activity buddies” (ie, “find health activity partners”). This produced 8 key phrase permutations with 30 results for each to give a total of 240 results.

Next, we identified emerging networks by searching TechCrunch and Y Combinator for the top 30 results from use of the common keyword “find” with the 2 stem terms “activity partners” and “activity buddies.” Prefix terms were excluded from the search to create a broader key phrase terminology with increased sensitivity to capturing emerging sites on TechCrunch and Y Combinator. This gave 2 key phrase permutations with 30 results for each of 2 start-up sites for a total 120 results. Together the searches produced a final list of 360 sites. Searches were conducted in September 2012.

For our review, we defined a “physical activity partner” as an individual who engages in physical activities offline with another user. Sites were defined as physical activity partner social networks if they had the ability to (1) actively find physical activity partners or activities for the user, (2) offer dynamic, real-time tracking or sharing of social activities, and (3) provide virtual profiles to users. We excluded from our analysis sites that were not Web-based, publicly available, in English, or free. Additionally, sites or applications that were mobile only or lacked a social network facet were excluded. The searches and determination of eligibility were conducted by 3 individuals (authors AN, AS, and RP) by using personal computers. Any discrepancies or conflicting opinions were brought before the group to reach a consensus.

### Analysis

We accessed and used each physical activity partner network to analyze site functionality and usability. A total of 12 key features (see Table 1 for descriptions of each feature) were assessed using a binomial scale (1=site has feature; 0=site does not have feature) for each site. Because there was sparse existing literature on which specific social network features would have an impact on physical activity, we looked to the existing sites themselves to determine which features were being used, developed, and incorporated into these sites with the goal of connecting physical activity partners to determine which features were important to analyze and characterize. Assessed features were placed into 1 of 3 categories: communication, activity optimization, and sophistication. Categories were picked to represent domains that would influence change in physical activity behaviors. The communication category broadly represented networks with features such as messaging, chat, or user updates. More specifically, this category included (1) ability to input or update status, (2) group creation, (3) private messaging, and (4) real-time messaging. The activity optimization category broadly represented networks with features allowing for tailoring of variables important in doing physical activity. This category included (1) ability to create activities, (2) activity creation customization, and (3) ability to directly confirm or join activities. The sophistication category broadly represented networks with advanced features that enhance user experience, interactivity, and value. This category included (1) exclusivity to physical activity, (2) filtering preferences, (3) interactive engagement prompts, (4) site-suggested recommendations for best fit activities, and (5) Web 2.0 technology integration (ie, with sites such as Facebook and Twitter).

**Table 1:** Description of key features and categories.

Category/feature name		Description
**Activity optimization**		Features that focus on the specifications of physical activity events that users can create or participate in
	Ability to create event	The website enables users to post or generate a new physical activity event
	Activity creation customization	Meets at least 5 of 9 predetermined variables relating to the user’s ability to customize activity creation: (1) the ability to customize according to specific activity type (eg, basketball), (2) time or day of event, (3) location, (4) event privatization, (5) invitation of individual participants, (6) invitation of groups, (7) skill level of participants, (8) maximum number of participants, and (9) the provision of a free response text box for further event information
	Direct ability to join event	Users may view physical activity events and have the option of selecting which events they intend on participating
**Communication**		Features that promote interactions between users
	Ability to input/update status	Users can submit text or media-based entries to describe their recent progress or activities
	Group creation	Users are able to create groups of common interest and have interactions within the group visible to all members of the group
	Private messaging of users	The website provides direct one-on-one messaging of users visible only to the users involved
	Real-time messaging (chat) of users	The website provides instant messaging for users to communicate directly with one another
**Sophistication**		Advanced features that support functionality of the site and are in place to lessen user burden connecting to the site and the physical activities offered
	Exclusivity to physical activity	The website has a singular focus on physical activities and does not specialize in connecting users based on nonphysical activities
	Filtering preferences	Meets at least 3 of 5 predetermined variables relating to the user’s ability to filter activities: (1) specific activity type, (2) user availability, (3) location, (4) user skill level, and (5) keyword
	Interactive engagement prompts	The website interacts with users through prompts such as questions about their physical activity interests or availability with the goal of connecting them to new activities
	Site-suggested best fit activities	The website offers activity recommendations for users based on previous site activity and collected user data
	Web 2.0 technology integration	The website has some connectivity involved with other social media sites, such as Facebook and Twitter

Of the 12 features, 3 were placed in the communication category, 4 were placed in the activity optimization category, and 5 were placed in the sophistication category. Of note, for the “activity creation customization” feature, a score of 1 was given if the site included at least 5 of 9 predetermined variables relating to the user’s ability to customize activity creation. The 9 predetermined variables were (1) the ability to customize according to specific activity type (eg basketball), (2) time or day of event, (3) location, (4) event privatization, (5) invitation of individual participants, (6) invitation of groups, (7) skill level of participants, (8) maximum number of participants, and (9) the provision of a free response text box for further event information. Additionally, for the “filtering preferences” feature, a score of 1 was given if the site included at least 3 of 5 predetermined variables relating to the user’s ability to filter activities. The 5 predetermined variables were (1) specific activity type, (2) user availability, (3) location, (4) user skill, and (5) keyword. The features were reviewed by 3 individuals (coauthors AN, AS, and RP) using individual personal computers. Each reviewer created an online account on each site and searched the site for the features of interest. All discrepancies among the reviewers were discussed until consensus was reached.

**Figure 1:**
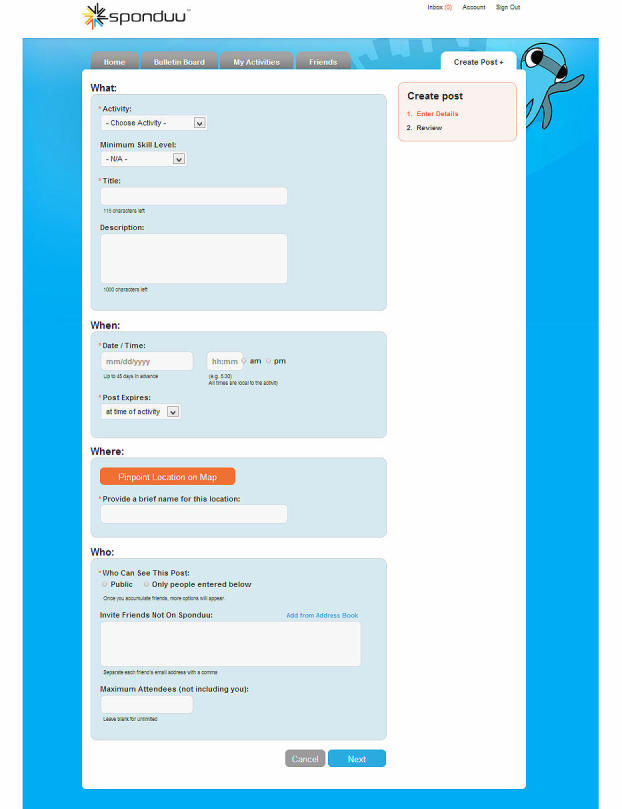
Sponduu: Ability to create event and activity creation customization.

**Figure 2:**
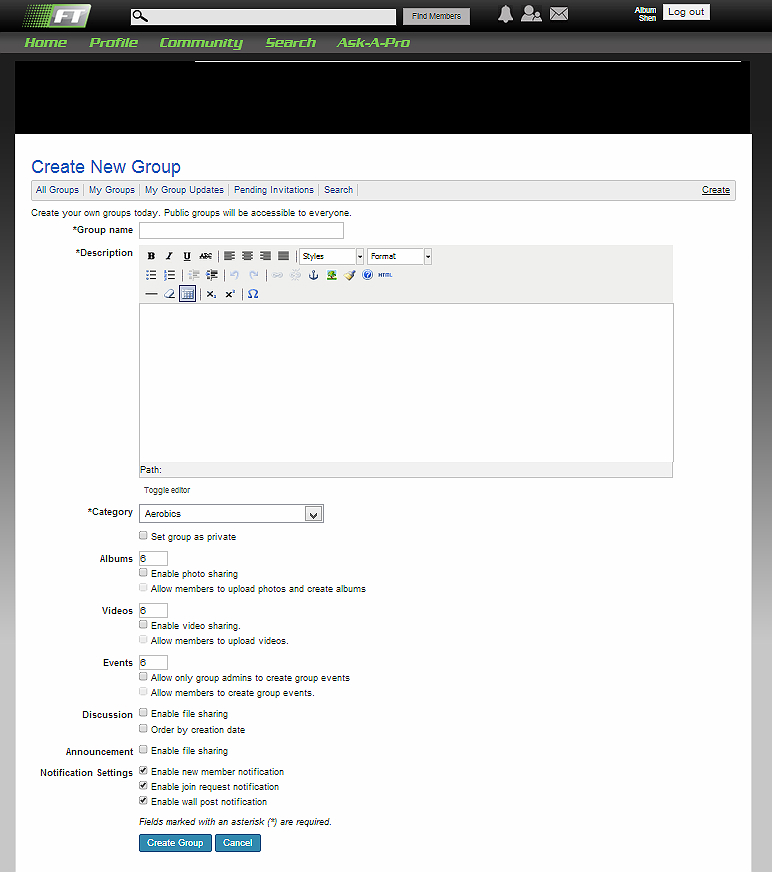
FitTogether: Group creation.

**Figure 3:**
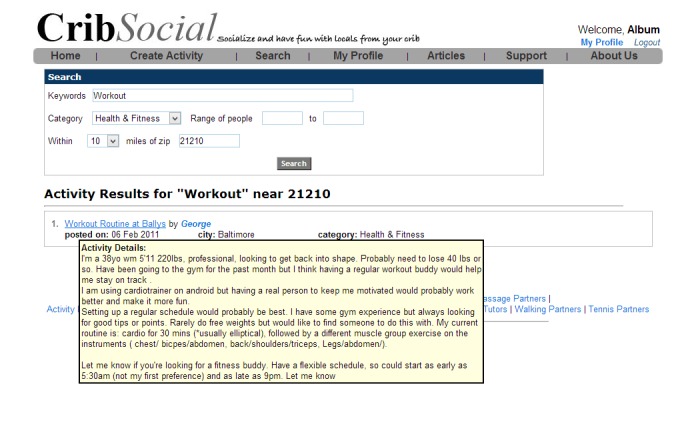
CribSocial: Filtering preferences.

**Figure 4:**
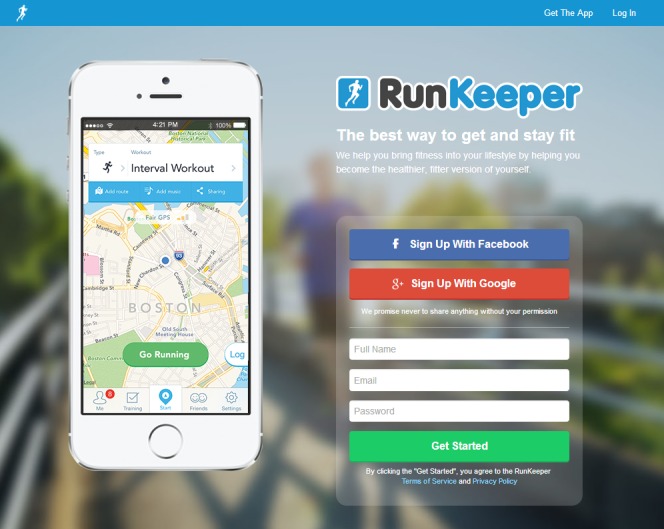
RunKeeper: Web 2.0 technology integration.

**Figure 5:**
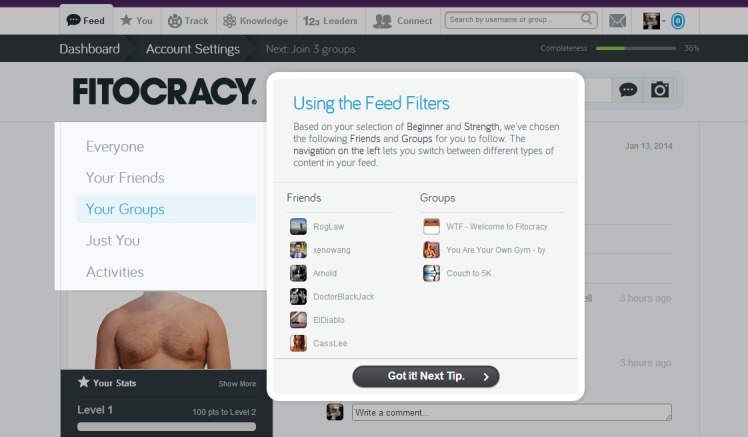
Fitocracy: Interactive engagement prompts.

Examples for some websites meeting the requirements for such features are represented in figures as follows. Sponduu meets the criteria for the ability to create event as well as activity creation customization by allowing users to create events and customize them with parameters that include activity type, skill level, title, free response text for description, date and time, location, event privatization, and invitation of individual participants (Figure 1). FitTogether offers group creation by allowing users to create groups that share photo albums, videos, events, and discussion visible to its users (Figure 2). CribSocial meets the filtering preferences criteria by allowing users to search for activities and filter by keyword, activity type, location, and number of participants (Figure 3). RunKeeper exhibits Web 2.0 technology integration by allowing users to sign up and sign in using their Facebook or Google accounts (Figure 4). Fitocracy offers interactive engagement prompts by asking the user for information regarding their interests in fitness, and recommends friends, groups, and activities that are relevant to their selected interests (Figure 5).

## Results

### Web Ranking

Of the 360 initial search results, we identified 13 established or emerging online social networks focused on physical activity. We arrived at these 13 sites after narrowing the search results by excluding results that were not social networks, that were repetitive or overlapping, or that were social networks but did not meet the criteria defined in our methods. The main characteristics of these websites are shown in Table 2, including the website name, worldwide website rank according to Alexa [10], and whether the site is established or emerging. The Web ranking measures a site’s popularity based on website traffic data; the lower the number, the higher the Web ranking and popularity. In 2012, the networks with the highest Web rankings were SparkPeople (2420), RunKeeper (5493), and Fitocracy (26,490).

**Table 2:** Description of websites for online social networks that connect users to physical activity partners.

Website name	2012 Web rank	Web presence
activity8 [11]	n/a	Emerging (beta)
BuddyUp [12]	3,138,866	Established
CribSocial [13]	1,246,680	Established
ExerciseFriends [14]	1,040,377	Established
Fitocracy [15]	26,490	Established
FitTogether [16]	358,988	Established
Friendeavor [17]	13,324,867	Established
Meet in Real Life [18]	5,556,996	Established
RunKeeper [19]	5493	Established
SparkPeople [5]	2420	Established
Sponduu [20]	12,274,504	Established
The Activity Partner [21]	15,847,153	Established
ZoomPal [22]	2,258,515	Emerging (beta)

### Distribution and Frequency of Features

Table 3 shows the frequencies of 12 key features across the networks, by whether they were prevalent (80%-100%), common (50%-79%), or rare (0-49%). These features are placed into 3 categories: communication, activity optimization, and sophistication. Features that appeared almost universally among these sites included the ability to create events (13/13, 100%), and private messaging (12/13, 92%). Common features among the sites were integration with Web 2.0 technologies, such as Facebook and Twitter (9/13, 69%), direct joining of activities (8/13, 62%), and group creation (8/13, 62%). Finally, highly sophisticated features were generally rare and often lacking on sites, such as filtering preferences (2/13, 15%), site-suggested best fit activities (3/13, 23%), and interactive engagement prompts that solicit user interaction (3/13, 23%).

Tables 4 and 5 display each of the 12 features (rows) for the 13 websites (columns) surveyed, along with the percent of features included in each website overall. The percentage of features that each website offered ranged from 25% to 75%. On average, the 13 social networks had 6.08 (SD 2.00) or 47% of 12 features assessed. The 2 social networks with the greatest percentage of the listed features were FitTogether and Fitocracy, each with 75% (9/12 features). Two other social networks, Sponduu and SparkPeople, had 58% or 7 of 12 features.

Among all sites, the activity optimization features category was best represented. On average, the sites included 67% (2/3) of the activity optimization category features, followed by 44% (1.77/4) of communication category features, and 37% (1.85/5) of sophistication category features.

**Table 3:** Features of online social networks categorized according to frequency (prevalent, common, or rare; N=13).

Frequency		Category	Frequency of feature among sites, n (%)
**Prevalent (80%-100%)**			
	Ability to create event	Activity optimization	13 (100)
	Private messaging of users	Communication	12 (92)
**Common (50%-79%)**			
	Web 2.0 technology integration	Sophistication	9 (69
	Direct ability to join event	Activity optimization	8 (62)
	Exclusivity to physical activity	Sophistication	7 (54)
**Rare (0-49%)**			
	Group creation	Communication	6 (46)
	Activity creation customization	Activity optimization	5 (38)
	Ability to input/update status	Communication	4 (31)
	Interactive engagement prompts	Sophistication	3 (23)
	Site-suggested best fit activities	Sophistication	3 (23)
	Filtering preferences	Sophistication	2 (15)
	Real-time messaging (chat) of users	Communication	1 (8)

**Table 4:** Features of online social networks that connect users to physical activity partners (A-F).

Feature		activity8	BuddyUp	CribSocial	Exercise Friends	Fitocracy	Fit Together	Friendeavor
**Activity optimization**								
	Ability to create event	Yes	Yes	Yes	Yes	Yes	Yes	Yes
	Activity creation customization	No	No	Yes	No	Yes	Yes	No
	Direct ability to join event	No	Yes	Yes	No	Yes	Yes	Yes
**Communication**								
	Ability to input/update status	Yes	No	No	No	Yes	No	Yes
	Group creation	No	Yes	No	Yes	Yes	No	Yes
	Private messaging of users	Yes	Yes	Yes	Yes	Yes	Yes	Yes
	Real-time messaging (chat) of users	No	No	No	No	Yes	No	No
**Sophistication**								
	Exclusivity to physical activity	Yes	Yes	No	Yes	Yes	No	Yes
	Filtering preferences	No	No	Yes	No	No	No	No
	Interactive engagement prompts	No	No	No	No	No	No	Yes
	Site-suggested best fit activities	No	No	No	No	No	No	Yes
	Web 2.0 technology integration	Yes	No	Yes	No	Yes	No	Yes
	% of Features	41%	41%	50%	33%	75%	33%	75%

**Table 5:** Features of online social networks that connect users to physical activity partners (M-Z).

Feature		MIRL	RunKeeper	SparkPeople	Sponduu	TheActivity Partner	ZoomPal
**Activity optimization**							
	Ability to create event	Yes	Yes	Yes	Yes	Yes	Yes
	Activity creation customization	No	No	Yes	No	No	Yes
	Direct ability to join event	No	Yes	Yes	No	No	Yes
**Communication**							
	Ability to input/update status	Yes	No	No	No	No	No
	Group creation	No	No	Yes	Yes	No	No
	Private messaging of users	Yes	Yes	Yes	Yes	Yes	No
	Real-time messaging (chat) of users	No	No	No	No	No	No
**Sophistication**							
	Exclusivity to physical activity	No	Yes	No	No	No	Yes
	Filtering preferences	No	No	Yes	No	No	No
	Interactive engagement prompts	No	Yes	No	No	No	Yes
	Site-suggested best fit activities	No	Yes	No	No	Yes	No
	Web 2.0 technology integration	Yes	Yes	Yes	No	Yes	Yes
	% of Features	33%	58%	58%	25%	33%	50%

## Discussion

### Principal Findings

In our investigation of 13 online physical activity social networks that connect users both online and face-to-face, we found that only half of these social networks contained more than 6 (50%) of the 12 distinct features we evaluated. Features related to activity optimization and communication were the most common among the social networks, whereas features related to sophistication were less common.

Today, more than ever, individuals are looking for easily accessible, low-cost, online technologies to address their health needs [23]. Currently, no particular website offers strong capabilities across the board in the areas of communication, activity optimization, and sophistication. This limits the extent to which these technologies may be successful in engaging individuals and, just as importantly, in fostering their behavior change.

Depending on the needs of individuals, particular sites may be of greater utility. SparkPeople and Fitocracy were the most sophisticated options and would suit individuals with low motivation levels who require a reduced-burden site and a more user-friendly experience. Although motivation and user-friendliness were not quantified in this analysis, the increased interactivity of these services might reflect users’ perceived effectiveness and value of the service [24]. A recent study tracked 1258 users of SparkPeople and found that those who were more active on the site (ie, by posting comments and messages to other users) saw the most significant weight loss outcomes [25]. For individuals with very specific activity needs, Sponduu offers the most tailored ability to optimize physical activity preferences by allowing users to create activities and customize them by using inputs such as activity type, time, location, skill level, privacy settings, maximum attendees, and a description text box. Additionally, its search feature allows users to filter results by using 149 physical or volunteering activities, location, and activity creator (friends, public, volunteer organization). The main advantage of such customization depth is that it takes into account user preferences, which include how rigorous the desired activities may be, and with whom the user would like to engage in the activity. Thus, such optimization reflects user needs, offering enhanced ability to interact with ideal target physical activity partners [26]. For individuals in need of communicative social support, MatchMySport and FitTogether provide an array of tools in the form of messaging, chat, and sharing of life updates. In addition to these features, certain websites, such as SparkPeople, also offered additional benefits such as calorie counting and diet tracking (ie, nutritional content beyond calories) features for users who are also interested in diet monitoring.

Out of the 13 websites evaluated in our research, FitTogether and Fitocracy had the highest percentage of the features at 75.0% or 9 of 12 features. However, FitTogether was particularly weak in the sophistication category, lacking 3 of 5 features (filtering preferences, interactive engagement prompts, site-suggested best fit activities). By lacking sophistication, user burden may be elevated to the point that engagement with the service is inconvenient and decreases over time. This is withstanding the notion that some simplistic platforms may provide lesser cognitive load and user burden. Here, we suggest that there is the possibility of further reducing user burden through use of more sophisticated software that aids in the automation of how social networks are used. This type of technological sophistication may operate on the backend such that users would only perceive the benefits of these algorithms or features without viewing added complexity to the interface [27]. Fitocracy may appear to have a more balanced collection of features than the other social networks by lacking just 1 item from each of the 3 categories (activity creation customization, real-time messaging, and filtering preferences), but among these missing features is the ability to create events that engage and connect people in their physical community. Fitocracy may not be able to sustain user commitment to regular physical activity as effectively without this option. Although it may not be impossible for users to meet with one another offline, it would be much more difficult for them to do so through the site.

Existing research has demonstrated both modest and significant gains in physical activity and health through the use of physical activity social network interventions [25,28-31]. Our study addresses the larger movement of catalyzing behavior change through online technologies and the need for scientific evidence. Although there exists no one-size-fits-all solution to combating physical inactivity today, the possibility of creating a scalable, low-cost, and universally accessible intervention now exists. Online social networks have already demonstrated effective behavior change in the areas of smoking cessation and safe sex practices [32]. Studies have shown that participants with access to an interactive computer program were likely to achieve higher smoking cessation rates [32]. Furthermore, study participants who used the program accompanied by a stop smoking forum were even more likely to retain progress than those with a less complete program [32]. An additional feasibility study has indicated that minimal contact/self-help interventions have yielded a 20.7% rate in 7-day cessation, and a 75% increase in participants’ reported intention to quit smoking [33]. What once required resource-intensive clinical management or elaborate public health strategy now may be possible with more simple technological aids.

### Strengths and Limitations

There are limitations to this work that are worth noting. First, we only evaluated websites that aimed to connect its users both online and face-to-face. We acknowledge that there are many ways to increase social support for physical activity without having to connect face-to-face, but these sites were not the focus of this descriptive work. Additionally, given our exclusion of certain networks from this analysis, we are limited in our ability to describe features for mobile apps that are not Web-based (eg, LoseIt!) or are sensor-driven networks (eg, Fitbit). Lastly, because of the descriptive nature of this work we are able to comment on the characteristics of sites, but are unable to comment on which of these characteristics actually engage users in physical activity. In principle, we analyzed features of websites rather than the effects of these sites on health. There are also several strengths of this work. First, these results may help guide health professionals faced with patients looking for online social support for their physical activity efforts. Additionally, it provides a snapshot of existing features of sites that aim to connect individuals both online and face-to-face for physical activity. Lastly, we generate hypotheses about what features of the online social networks reviewed might be helpful in initiation and maintenance of physical activity.

### Clinical Implications

Previous studies suggest that online social networks are good platforms for intervention delivery especially among young adults [34,35]. There is evidence that features such as tailored content and goal setting assists in promoting the effectiveness of physical activity interventions [36]. Further, it has been hypothesized that interventions may yield more favorable outcomes with the use of advanced features, such as automated dialog and more personalized forms of communicating information [37]. Participant dropout from physical activity intervention programs has been a notable problem in this area of research [38]. Certain features, such as email reminders, supervision and contact through texting, and regularly updated content, might be harnessed to help with adherence [39].

A major concern in this quickly growing area of research is the extent to which technological creation is quickly outpacing scientific evidence. Online services are adopted at a blazing pace. For example, SparkPeople and RunKeeper were used by over 15 million and 23 million users, respectively [5,6].

### Moving Forward

The objective of this descriptive analysis was to characterize the existing features of sites in play that connect users with physical activity partners both online and face-to-face. In principle, this analysis allows us to generate hypotheses about why certain sites may help individuals initiate or maintain physical activity behaviors, but it does not provide information on the effect of these sites on physical activity or health. Moving forward, research is needed to evaluate the impact of these features on physical activity given the need for empirical foundations for the continued use or elimination of features. Currently, viral word-of-mouth and popularity impacts adoption [40,41], whereas ideally this would be driven by data. The use of a site’s popularity to increase user adoption is a precarious path for those looking for genuine evidence-based health care interventions because popular programs (eg, video games) have been shown to be ineffective in promoting physical activity [42].

We found that the distribution of features on each site varied widely, with no single site including 100% of the features we reviewed. Although it is not necessary to include every feature to be effective in promoting physical activity, as a next step, it is worth generating evidence about which features are most important to users and which features are most effective in promoting physical activity. It has been suggested that online social networks makes forming groups easier than it has even been, that there can be either positive or negative effects of the easy collaborative nature of online social networks, and that a that a critical mass of individuals is required for social networks to be useful [43-45]. Moving forward, we suggest additional research to explore several hypotheses. For example, we would hypothesize that features such as communication, activity optimization, and sophistication might be key contributors to behavior change. We would also hypothesize that if sites had more engaging user interfaces, including easier navigation, simpler layouts, and refined esthetics, there would be greater initiation of use and physical activity. Lastly, we would hypothesize that the features we identified could potentially be used to overcome the challenge of requiring a critical mass of individuals for social network sites to be useful. Although it is promising to see the development and availability of public physical activity social networks, research is needed that includes other networks, such as those that are Web-based or sensor-driven, as well as research that discerns which tools can offer meaningful behavioral impact and guide effective public health policy and clinical counseling. Many questions remain around how users utilize these services and how helpful they truly are.

## Abbreviations

URL: uniform resource locator
